# Urinary metabolomics reveals the therapeutic effect of HuangQi Injections in cisplatin-induced nephrotoxic rats

**DOI:** 10.1038/s41598-017-03249-z

**Published:** 2017-06-15

**Authors:** Chang-Yin Li, Hui-Ting Song, Xiao-Xiao Wang, Yao-Yao Wan, Xuan-Sheng Ding, Shi-Jia Liu, Guo-Liang Dai, Yue-Heng Liu, Wen-Zheng Ju

**Affiliations:** 1 Department of Clinical Pharmacology, Affiliated Hospital of Nanjing University of Chinese Medicine, No. 155 Hanzhong Road, Nanjing, 210029 China; 2 School of pharmacy, China Pharmaceutical University, No. 639 Longmian Road, Nanjing, 211198 China; 3 School of pharmacy, Nanjing University of Chinese Medicine, No. 138 Xianlin Road, Nanjing, 210023 China

## Abstract

The side effects of cisplatin (CDDP), notably nephrotoxicity, greatly limited its use in clinical chemotherapy. HuangQi Injections (HI), a commonly used preparation of the well-known Chinese herbal medicine *Astragali radix*, appeared to be promising treatment for nephrotoxicity without compromising the anti-tumor activity of CDDP. In this study, the urinary metabolomics approach using liquid chromatography time of flight mass spectrometry (LC-TOF/MS) was developed to assess the toxicity-attenuation effects and corresponding mechanisms of HI on CDDP-exposed rats. As a result, successive administration of HI significantly recovered the decline of body weight and downregulated the abnormal increase of serum creatinine and urea. HI partly restored the CDDP-induced alteration of metabolic profiling back into normal condition. Totally 43 toxicity-attenuation potential biomarkers were screened and tentatively identified, which were involved in important metabolic pathways such as amino acid metabolism, TCA cycle, fatty acid metabolism, vitamin B6 metabolism and purine metabolism. The results clearly revealed that HI could alleviate CDDP-induced nephrotoxicity and improve the disturbed metabolic balance induced by repeated CDDP exposure. The present study provided reliable evidence for the protective effect of HI on CDDP-induced toxicity with the multi-target pharmacological characteristics.

## Introduction

Nephrotoxicity or renal toxicity has grown rapidly in the past ten years. As a holistic approach reflecting comprehensive changes of endogenous metabolites in bio-fluids and tissues, metabolomics has been widely applied to the study of the pathogenesis of nephrotoxicity or kidney diseases and discover new biomarkers as well as effect of drug intervention and therapeutic mechanism^1–4^. Metabolomic method was widely used to explore the nephrotoxicity induced by chemical drugs^5, 6^ and natural products^7–10^. Traditional Chinese medicines (TCMs) have been used for the treatment of different kidney diseases for thousands of years. TCMs have gained increasing attention for intervention of nephrotoxicity based on their long-standing and wide-spread clinical application^11^. Metabolomics-based studies have demonstrated bioactivity and biochemical action mechanism of *Rheum officinale* on nephrotoxicity^12–14^. The studies demonstrated that rhubarb may reverse up-regulation expression of collagen I, fibronectin, α smooth muscle actin and plasminogen activator inhibitor-1 proteins against renal fibrosis. The renoprotective effects of the layer of *Poria cocos* were systematically studied by using metabolomics approach^15–17^. These studies based on metabolomics technique demonstrated TCMs had a beneficial effect on treatment of kidney disease and highlighted the biochemical action mechanism of anti-tubulointerstitial fibrosis.

*Radix Astragali* (RA), a famous herbal medicine named as HuangQi in Chinese, is derived from the roots of *Astragalus membranaceus* (Fisch.) Bge. Both clinical and preclinical studies have demonstrated that RA possessed remarkable renoprotective activity^18, 19^ and antitumorigenic potential^20^. Therefore, RA preparations should be a promising treatment for nephrotoxicity without compromising the anti-tumor activity of cisplatin (CDDP). Proton nuclear magnetic resonance spectroscopy and mass spectrometry were widely applied to metabolomics study. Liquid chromatography-mass spectrometry (LC-MS) applied to metabolomics is one of the best analytical techniques in selectivity, sensitivity and reproducibility^21–24^. The earlier reports demonstrated docosahexaenoic acid, docosapentaenoic acid, tryptophan, phenylalanine, p-cresol sulfate and indoxyl sulfate were the biomarkers of nephrotoxicity and these biomarkers were closely associated with fatty acid metabolism, amino acid metabolism and energy metabolism^25–27^. The objective of the present study is to investigate the toxicity-attenuation effect of the commonly used RA preparation HuangQi Injections (HI) in the CDDP-exposed rats using LC-TOF/MS-based urinary metabolomics method.

## Materials and Methods

### Materials

HI (lot 1410263, 10 mL per bottle) preparation was provided by ChiataiQingchunbao pharmaceutical Co., Ltd. (Hangzhou, China). Using the LC-MS-MS method, the concentrations of five major constituents in HI were determined as follows: astragaloside IV, 119.4 µg/mL; calycosin-7 O-β-D-glucoside, 271.0 µg/mL; calycosin, 35.16 µg/mL; ononin, 85.55 µg/mL; formononetin, 8.718 µg/mL. Meantime, spectrophotometry analysis indicated that the concentration of polysaccharides was 983.6 µg/mL.

CDDP for injection (lot 2WA2A1307047B, 20 mg per bottle) were purchased from Qilu pharmaceutical Co., Ltd. (Hainan, China). Methanol and acetonitrile were of HPLC grade (Merck, USA). MS grade formic acid (lot BCBK9295V, purity: 98%) and ammonium formate (lot BCBK2322V, purity: ≥99%) were obtained from Fluka-Sigma-Aldrich Chemie (Steinheim, Switzerland). The assay kit for creatinine (Crea), crea and albumin were purchased from Beckman Coulter Commercial Enterprise (China) Co., Ltd. Ultrapure water was obtained in the laboratory by using the Milli-Q water purification system (Millipore, USA). APCI positive (PN. 4460131, Lot A4328) and negative (PN. 4460134, Lot A4287) calibration solutions for the AB SCIEX Triple TOF^TM^ system were obtained from the AB Sciex Pte. Ltd., USA.

### Animal experiment and sample collection

A total of 24 male SD rats (200–240 g) were randomly divided into 3 groups equally including untreated control rats (Control group), CDDP-exposed rats (CDDP group) and HI-treated CDDP-exposed rats (CDDP + HI group). Before experimentation, all the rats were acclimated for 7 days. During the whole experiment, they were maintained in a 12-h light/dark cycle in a temperature (22–24 °C) and humidity (45–55%) controlled facility. Standard chow and water were provided ad libitum.

On day 1, 3, 7, 11, 14, CDDP with the daily dosage of 2.5 mg/kg, or its vehicle was administered by intraperitoneal injection to rats in the same manner and volume (0.5 ml/100 g). From the first day of CDDP administration, HI was administrated to rats at 0.5 ml/100 g once daily (equivalent to the largest clinical recommended daily dose) for 16 consecutive days. Urine sample of each rat was collected over a 24-h period on day 18. After recording the total volume, 3 mL of each urine sample were divided into 2 aliquots for biochemical and metabolomics analysis. On day 20, all the rats were sacrificed to collect blood samples for biochemical analysis; meanwhile, kidney tissues were removed fixed in 10% neutral-buffered formalin for hematoxylin and eosin (H&E) staining. The serum samples were obtained by centrifugation of the whole blood of rats at 4000 *g* for 10 min at 4 °C. All urine and serum samples were stored at −80 °C prior to analysis. The animal experiments were performed according to the Guide for the Care and Use of Laboratory Animals and were authorized by the Animal Ethics Committee of Nanjing University of Chinese Medicine.

### Measurements of biochemical parameters and histopathology

The levels of serum urea and crea, urine crea and albumin excretion were measured using the Beckman Coulter AU5800 Clinical Chemistry Analyser (Beckman Coulter, USA). The glomerular filtration rate (GFR) was calculated as Csc × Vu/Cuc/24/60; of them, Csc and Cuc refers to the crea concentration of serum and urine, Vu refers to the volume of 24 h urine. After fixed overnight in 10% neutral-buffered formalin, kidneys were dehydrated in alcohol and then embedded in paraffin for tissue sectioning. The tissue sections (4 μm) were underwent hematoxylin-eosin (H&E) staining for structural evaluation by light microscopy at 200× magnification.

### Urine sample preparation

Before LC-MS analysis, all the urine samples were thawed and vortexed at room temperature and 200 μL aliquots of each sample were diluted with 300 μL of water and 500 μL of methanol. The mixture was vortexed for 30 s and subsequently centrifuged at 12000 *g* for 5 min at 4 °C. Finally, 5 μL aliquot of the supernatant was injected into the LC-TOF/MS system for analysis. A pooled quality control (QC) sample was made by mixing aliquots of the supernatant from each sample.

### LC-MS conditions

Urine chromatographic separation was performed on a series 1200 HPLC system (Agilent, USA) equipped with an Agilent poroshell 120 SB-C18 column (3.0 mm × 100 mm, 2.7 μm) and an Agilent poroshell SB-C18 guard column (3.0 × 5 mm, 2.7 μm). Ultrapure water (A) and acetonitrile/methonal (*v/v*, 1/1, B), both containing 0.1% formic acid (v/v) and 2 mM ammonium formate (*v/v*), were used as the mobile phase. Under the flow rate of 0.3 mL/min, the urinary metabolites were eluted with a gradient program as follows: 0–0.5 min, 5% B; 0.5–12 min, 5–100% B; 12–16 min, 16–16.1 min, 100–5% B; 16.1–22 min, 5% B. The column oven temperature was maintained at 35 °C, and all the samples were maintained at 8 °C during the whole analysis. The eluant was switched into waste channel for 0–1.3 min and 16.5–22 min and MS channel for 1.3–16.5 min by a Valve Valco 2-Position.

The Triple TOF^TM^ 5600 (AB SCIEX, Foster City, CA) equipped with electron spray ionization source was used for MS detection, and both positive and negative ion modes were employed. The ion spray voltage floating was set at 5500 V and −4500 V for positive and negative ion mode respectively. TOF/MS scan conditions were as follows: TOF mass range was set at *m/z* 100–1000; accumulation time: 0.25 s; ion source gas 1: 60 psi; ion source gas 2: 60 psi; curtain gas: 35 psi; heater temperature: 550 °C; declustering potential: 80 V; collision energy 10 eV; The options of IDA, DBS and high sensitivity were chosen. Major IDA switch criteria were as follows: Intensity exceeds 500 cps, exclusion isotope within 4 Da, mass tolerance 50 mDa, maximum number of candidate ions to monitor per cycle 8. For Product Ion scan type, TOF mass range was set at *m/z* 50–1000, accumulation time was set at 0.100006 s, collision voltage set at 35 ± 15 eV, ion release delay at 67 ms, ion release width at 25 ms, and the other parameters were same with TOFMS scan type. All the operations and acquisition were controlled by Analyst® TF 1.6 software (AB SCIEX, Foster City, CA).

### LC-MS analysis of urine samples

Prior to real sample analysis, the pooled QC sample was analyzed in 10 replicates to precondition the analytical system at the beginning of the analytical run, and then injected every 10 urine samples through the analytical run to monitor the stability of the analytical system. All rat urine samples in different groups were analyzed randomly to remove any deviation from the injection order. The mass accuracy of the detection was auto-calibrated every 5 samples using the Calibrant delivery system (CDS).

### Validation of analytical method

QC samples inserted in the real-sample analysis were used to validate the LC-TOF/MS analysis of urine samples, or in other word, to assess the repeatability and stability of the analytical system^28^. First, typical ions from the LC-TOFMS data sets of the QC samples were randomly picked only when they were widely distributed in different *m/z* and retention time (*t*_R_) and corresponding to the regular peak shapes. Then, three values of the typical ions including *m/z*, *t*_R_, and ion intensity, were obtained using the XIC manager of Peak view software 1.2.0 (AB SCIEX, Foster City, CA). The shifts of *m/z* and *t*_R_, as well as the relative standard deviation (RSD%) of ion intensity were calculated to evaluate the reproducibility of the typical ions. Meanwhile, the LC-TOFMS data sets of the QC samples were imported into Simca-P software 14.0 (Umetrics, Sweden) for principal component analysis (PCA), to clarify whether all QC samples were clustered closely in the corresponding PCA score plots.

### Data analysis

#### Data preprocessing

The raw LC-TOFMS data obtained from LC-MS analysis of all the samples were processed using MarkerView v1.2.1 software (AB SCIEX, Foster City, CA). Pre-treatment procedures including peak finding, alignment, filtering and normalization were performed to process the raw data. The major data extraction parameters were as follows: *t*_R_ range 1.3–16.5 min, minimum spectral peak width 25 ppm, minimum *t*_R_ peak width 6 scan, mass tolerance 20 ppm, *t*_R_ tolerance 0.3 min, noise threshold 100, maximum number of peaks 8000, excluding isotopic peaks. The ion intensities from each urine sample were normalized by the MS Total Useful Signals (MSTUS)^29^, with the purpose of correcting the variations in urine volumes excreted per time. The resulting three dimensional matrixes were composed of sample names, *m/z* and *t*_R_ pairs, and normalized ion intensities. The “80% rule” was employed to remove missing peaks^30^. The data was then subjected to multivariate data analysis (MVDA) and statistics.

#### Multivariate data analysis and statistics

The preprocessed data set was imported into the SIMCA-P 14.0 to perform MVDA. After pare to scaling, the data were subjected to the unsupervised PCA and supervised orthogonal partial least squares-discriminant analysis (OPLS-DA). PCA was used to assess the stability of the analytical system and the quality of the data set, and to detect the possible outliers. While OPLS-DA was employed to obtain an overview of the data of all samples, and clarify the varied metabolites responsible for the discrimination between the groups. The quality of the OPLS-DA models was evaluated with the relevant R^2^ and Q^2^,^31^ while permutation test was utilized to assess the predicative ability of the models and their statistical significance. The scores of Variable Importance in Projection (VIP), derived from OPLS-DA, were used to screen the variables relevant for group discrimination. Meanwhile, the two-tailed independent Student’s t-test and fold change (FC) analysis were also applied to evaluate the significant differences in the variables using the Marker View software. Compared to Group CDDP, The differential metabolites with the same change trend in Group Control and Group CDDP + HI were selected as potential biomarkers (PBs) of CDDP-toxicity attenuation when they meet the following 3 criteria simultaneously: VIP > 1, *p* < 0.01 and FC > 2.

#### Metabolite identification and metabolic pathway analysis

The PBs for CDDP-toxicity attenuation were mainly identified based on their LC-TOF/MS data. First, with mass tolerance of less than 5 mDa, the quasi-molecular ions of the PBs and their fragment ions in product ion type were matched with that of metabolites collected in several online related databases including METLIN (http://metlin.scripps.edu/), HMDB (http://www.hmdb.ca/), KEGG (http://www.kegg.com/), Mass Bank (http://www.massbank.jp/), Chemspider (http://www.chemspider.com/) and PubMed (http://www.ncbi.nlm.nih.gov/). If there was no fragment ion information in the databases, the possible structures of the PBs were imported into Peakview software, and then fragmented theoretically by the fragments pane integrated in the software, and finally matched with the experimental MS/MS spectra. Only when both the quasi-molecular ions and fragment ions were well matched with the known metabolite in the database with good mass accuracy (<5 mDa), the corresponding PB was considered to be tentatively identified.

**Figure 1:**
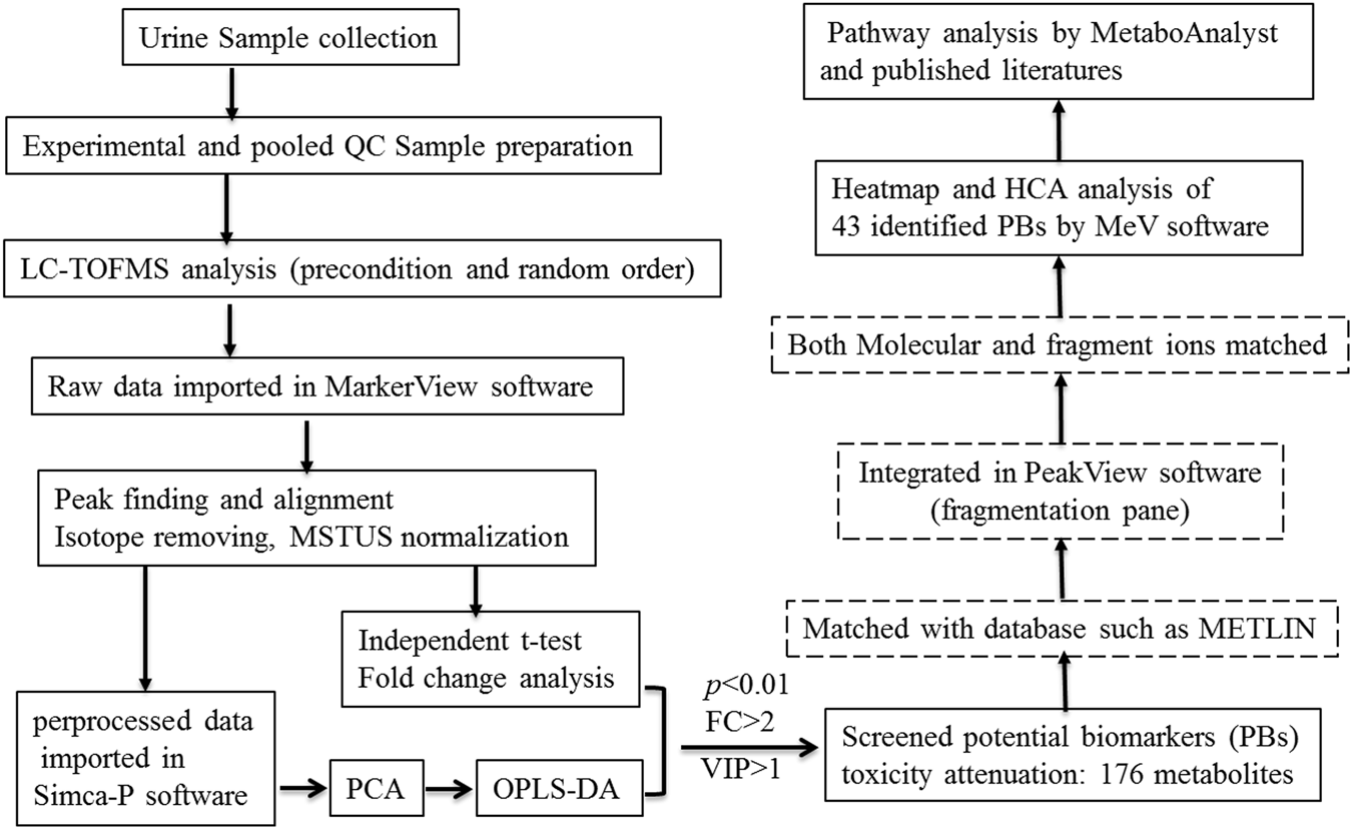
LC-TOF/MS based roadmap for the urinary metabolomics study on toxicity-attenuation effect of HuangQi Injections in CDDP-exposed rats.

The dataset of the identified PBs was imported into the MeV software package (version 4.6.0) for Heatmap generation and hierarchical cluster analysis (HCA). In order to explore the molecular mechanism of CDDP-toxicity attenuation of HI, pathway analysis of the dataset of identified PBs was performed using MetaboAnalyst (http://www.metaboanalyst.ca/)^32^. In addition, the literatures published were extensively searched to clarify the biological functions or meanings of all the identified PBs. Figure 1 displayed a roadmap from sample collection to data analysis and metabolite identification.

## Results

### General pharmacological assessment

**Figure 2:**
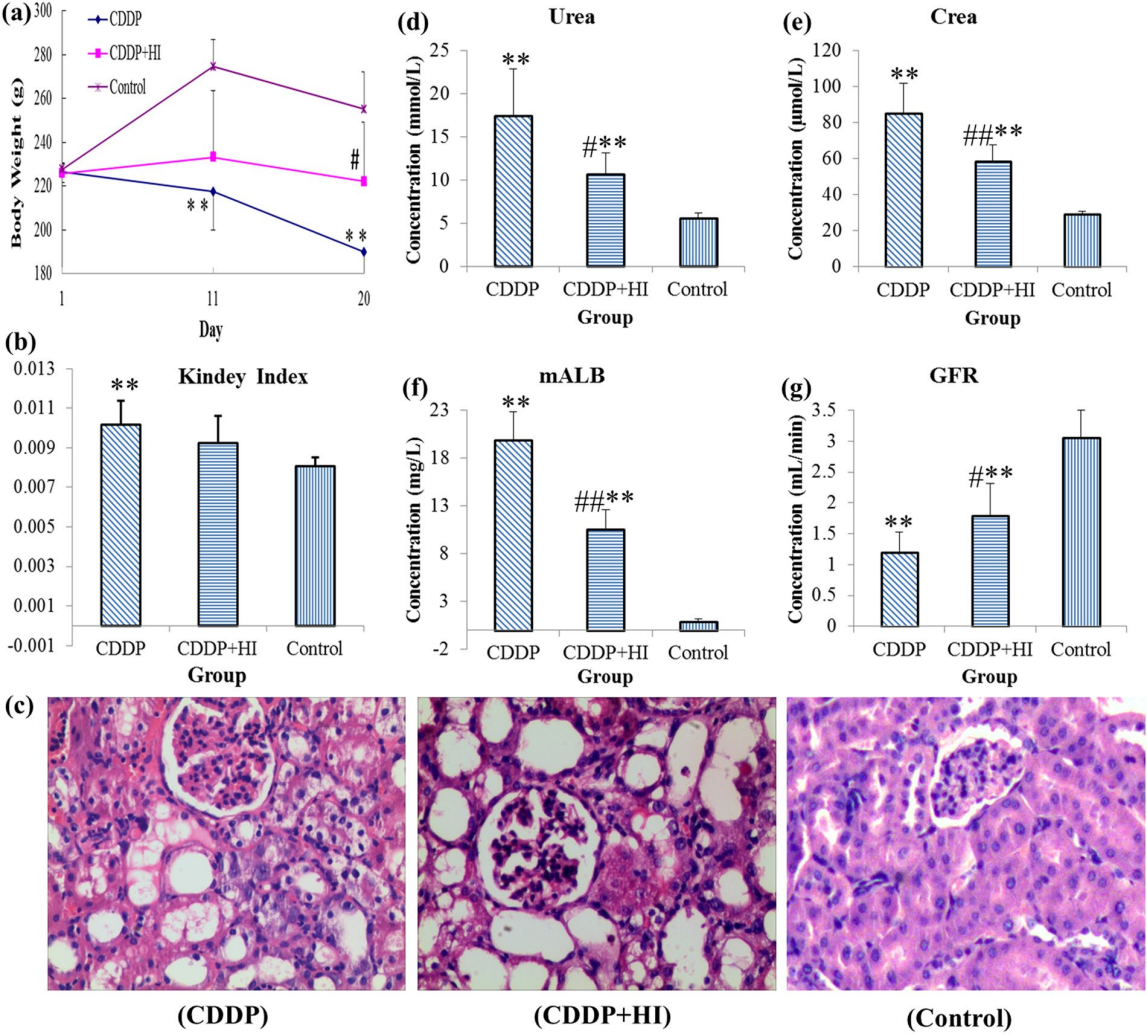
The general pharmacological effects of HuangQi Injections on CDDP-exposed rats (*n* = 8). (**a**) Body weight, (**b**) kidney index, (**c**) hematoxylin-eosin staining histological appearance (Magnification × 200) of kidney, (**d**) serum urea level, (**e**) serum crea level, (**f**) urinary mALB level, (**g**) glomerular filtration rate (GFR). Compared with Control, *p < 0.05, **p < 0.01; compared with CDDP, ^#^p < 0.05, ^#^p < 0.01.

Figure 2a shows the influence of HI on the body weights (BWs) of CDDP-exposed rats on day 1, 11 and 20. It can be seen that remarkable decline of BWs in Group CDDP were observed on day 11 and 20, while successive administration of HI for 16 days could significantly recover this decline. As shown in Fig. 2b, the index of kidney in Group CDDP were significantly higher than that in Group Control, and HI administration could ameliorate this change to some extent, with no statistical significance. In addition to the above the macroscopic indicators, the microscopic morphology of kidney were also significantly changed after cisplatin administration. Figure 2c showed the representative pathological examination results of kidney in different groups. No abnormal changes were observed in Group Control. Remarkable abnormal histological changes can be observed in Group CDDP, and the damages manifested as tubular necrosis, tubular expansion, and interstitial infiltration of inflammatory cells. Group CDDP + HI shows some improvement of the above histological damages compared to Group CDDP. Urea, Crea, urine albumin excretion and GFR are commonly used clinical biochemical parameters for the evaluation of renal function^31^. As shown in Fig. 2d,e,f and g, after repeated administration of CDDP, the levels of serum Urea, serum Crea and urinary mALB were significantly upregulated (*p* < 0.01), while GFR level was significantly downregulated. Successive administration of HI could significantly improve the above abnormal changes (*p* < 0.05 for serum Urea and urinary mALB, *p* < 0.01 for serum Crea and GFR). Collectively, the results of BW changes, kidney index, histological examination and biochemical parameters clearly confirmed the presence of CDDP-induced nephrotoxicity, as well as the reno-protection of HI.

### Analytical method validation

**Figure 3:**
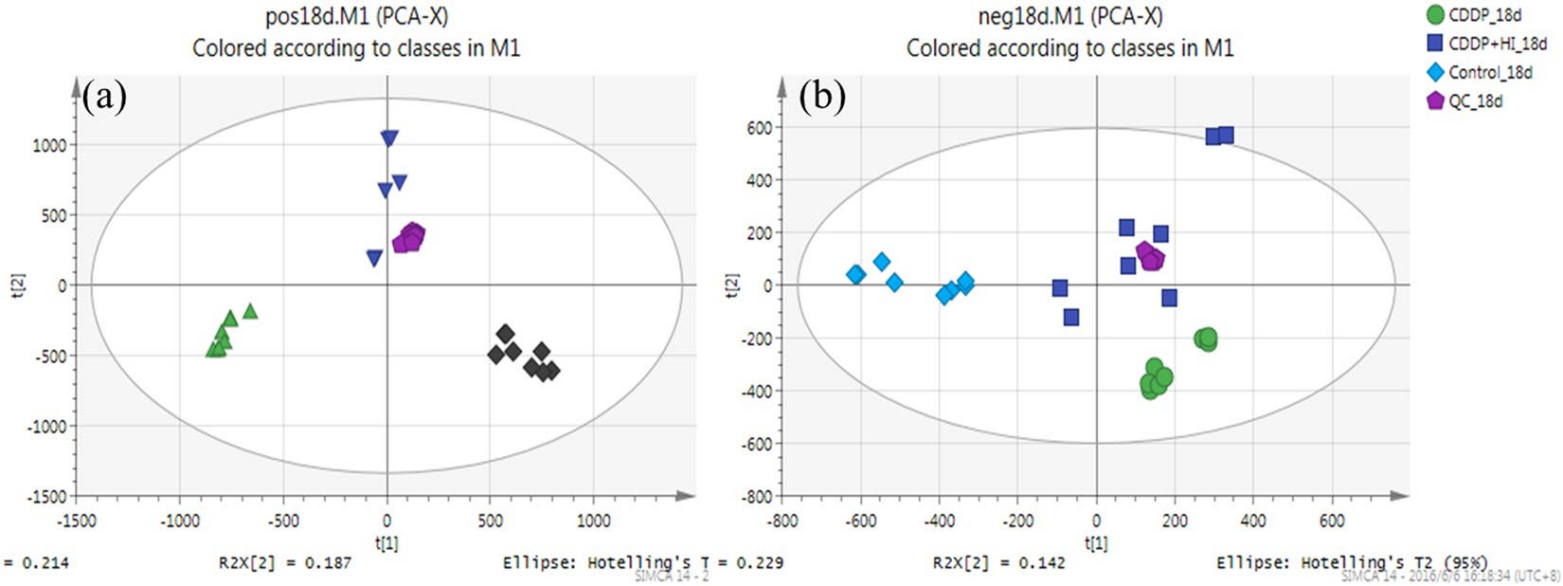
PCA score plots of metabolic profiling of urine samples in positive ion mode (**a**) and negative ion mode (**b**).

In LC-TOF/MS-based metabolomics study where large sample batches are always involved with, key characteristics of the analytical system, both chromatographic and mass spectrometric (e.g., retention time stability, peak shape, detector response and mass accuracy), may gradually change over the analysis time. Therefore, simple and effective measures should be taken to rapidly assess the obtained LC-MS data quality. In this study, the use of QC samples, a widely used approach for ensuring the validity of global metabolic profiling data^28^, was employed for the validation of the LC-TOFMS analysis. The stability of the three features of the analytical system including *t*_R_, signal intensity and mass accuracy was investigated by 16 typical ions of 5 within-run QC samples in both positive and negative ion modes. As shown in Table S1, the maximum *t*_R_ shifts in positive and negative ion modes were 0.21 min and 0.15 min respectively, the maximum mass deviations were 5.84 ppm and 6.98 ppm, while the RSD% of signal intensity ranged from 1.87 to 9.88% in positive ion mode, 3.01–9.52% in negative ion mode. The results indicated that the analytical system has good stability during the whole batch analysis. PCA analysis further validated the reproducibility of analytical method. As shown in Fig. 3, five within-run QC samples were closely clustered with each other in the PCA score plots in both positive and negative ion modes.

### Multivariate analysis of the metabolic profiles

**Figure 4:**
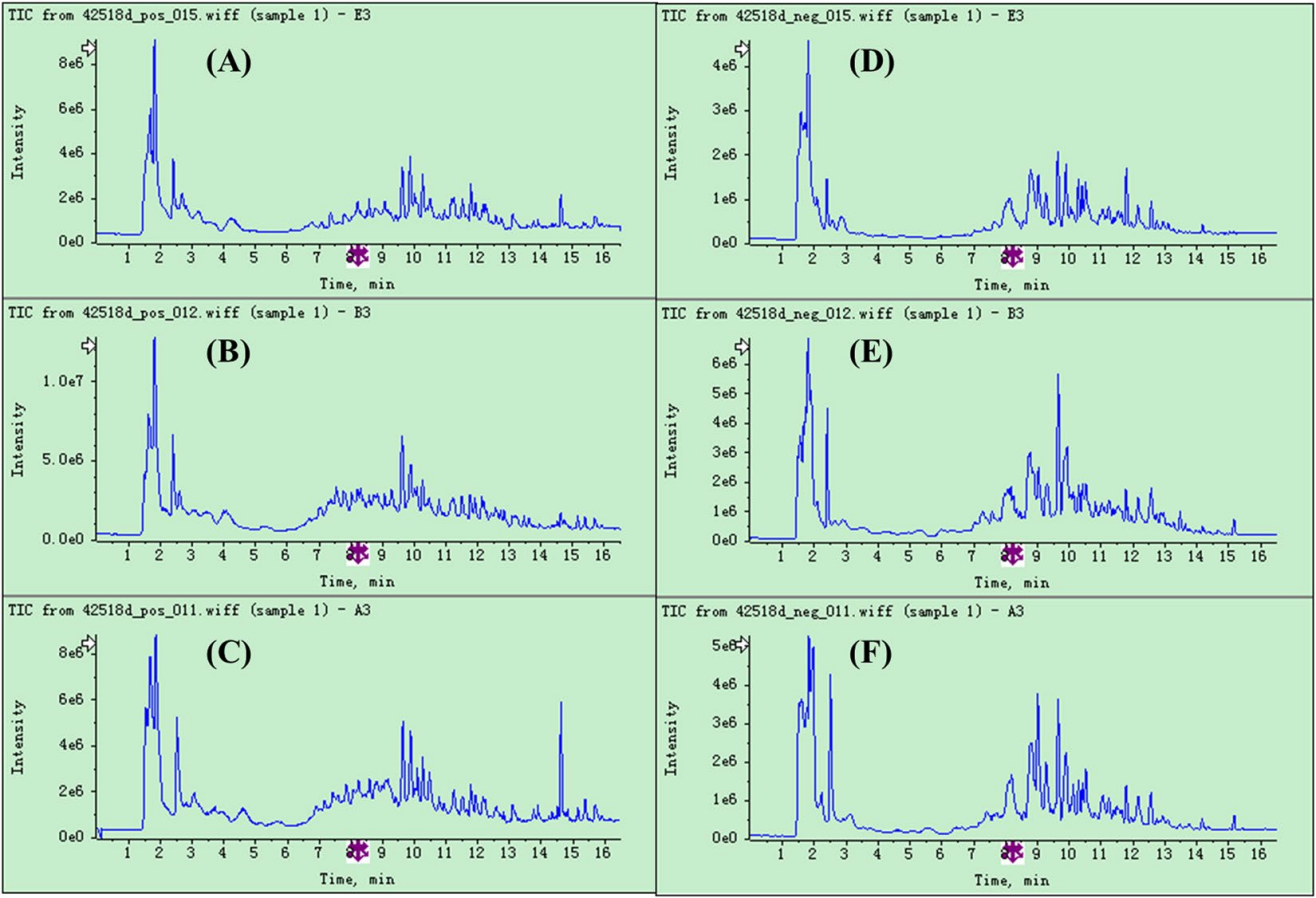
Representative LC-TOF/MS total ion chromatograms (TICs) of rat urine samples from Control (**A**), CDDP + HI (**B**) and CDDP (**C**) groups in positive ion mode, and Control (**D**), CDDP + HI (**E**) and CDDP (**F**) groups in negative ion mode.

Representative LC-TOF-MS total ion chromatograms (TICs) of rat urine samples from all 3 groups in positive and negative ion modes are shown in Fig. 4, from which some difference in metabolic profiles of different groups could be observed. In order to better visualize the similarities and differences among these complex data sets, PCA and OPLS-DA analysis were used to classify the metabolic phenotypes and identify the differentiating metabolites. As shown in the PCA score plots (Fig. 3), the urine samples within each group were closely clustered into each other in both positive and negative ion mode, while samples from different groups were clearly separated. Meanwhile, data in positive ion mode showed better clustering and separation than that in negative mode. Group CDDP + HI was located in the middle of Group CDDP and Group Control, indicating the remarkable CDDP-induced toxicity and obvious toxicity-attenuation effect of HI from the perspective of metabolic profiles.

The results of OPLS-DA analysis including score plots, S-plots and chance permutation tests between Group CDDP and Group Control or CDDP + HI are presented in Fig. S1. The relevant R2Y and Q2Y were utilized to evaluate the quality of the OPLS-DA models: R2Y represents the goodness-of-fit parameter of the OPLS-DA model, while Q2Y represents the predictability parameter of the OPLS-DA model. Generally, a Q2Y > 0.5 is regarded as good and a Q2Y > 0.9 as excellent, while differences between R2Y and Q2Y larger than 0.2–0.3 indicate the presence of many irrelevant model terms or a few outlying data points. As shown in Fig. S1A–D, in all the OPLS-DA models, the values of Q2Y were no less than 0.9, and the difference between R2Y and Q2Y was no more than 0.2, indicating excellent quality of our models. From the S-plots of OPLS-DA model (Fig. S1E–H), it can be seen that hundreds of differential metabolites (VIP > 1, marked with red) were responsible for the apparent discrimination in every OPLS-DA model. Permutation test was utilized to assess the predicative ability of the OPLS-DA models and their statistical significance. In the permutation test, experience shows that the Q2Y-intercept should not exceed 0.05. As shown in Fig. S1I–L, all Q2Y-intercepts meet this experience criterion, demonstrating that our OPLS-DA models were well validated. The differential metabolites derived from OPLS-DA model were then filtered by independent t-test and FC analysis to remove any false differential metabolite. Potential biomarkers (PBs) between Group CDDP and Group Control or CDDP + HI were finally selected following the criteria of VIP > 1, *p* < 0.01 and FC > 2. According to their change trends among 3 groups, totally 176 toxicity-attenuation PBs of HI treatment were picked out for further identification.

### Potential biomarker identification

**Figure 5:**
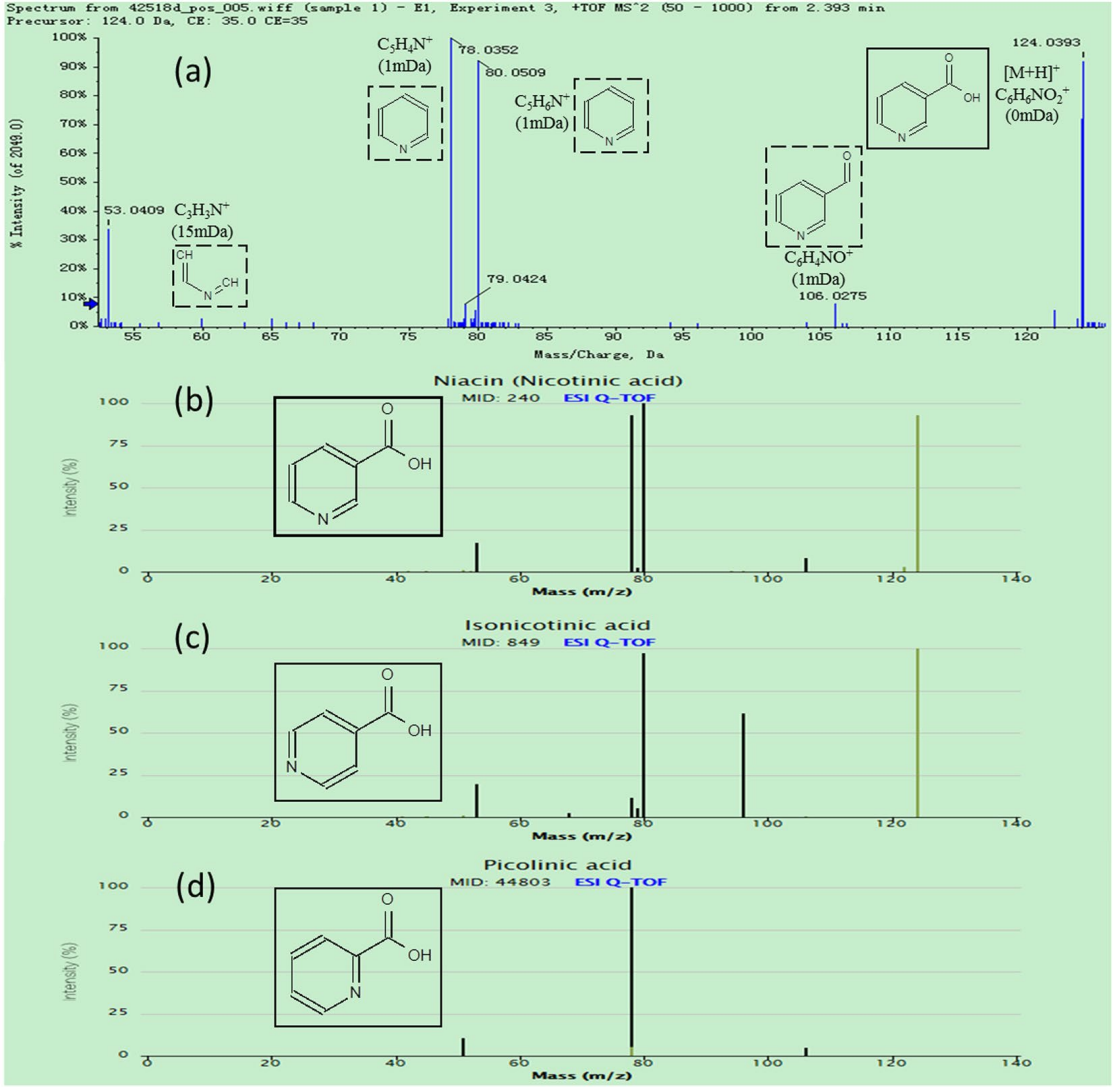
Data for the identification of nicotinic acid (P13): (**a**) The product ion spectrum of PB13 in positive ion mode; (**b**) MS/MS spectrum of nicotinic acid in Metlin database; (**c**) MS/MS spectrum of isonicotinic acid in Metlin database; (**d**) MS/MS spectrum of picolinic acid in Metlin database.

Using the identification strategy, the tentative identification of all 176 PBs in urine samples were performed according to their mass spectra information in TOFMS and Product Ion scan types. **PB13** with *m/z*_*t*_R_ of 124.0398_2.39 in positive ion mode was taken as an example to illustrate the identification process. Firstly, according to the accurate measurement of quasi-molecular ion at *m/z* 124.0398 within mass tolerance of 5 mDa, 4 candidate compounds with the same elemental composition of C_6_H_5_NO_2_ were found in METLIN database, including nicotinic acid, isonicotinic acid, picolinic acid and nitrobenzene. As shown in the experimental product ion spectrum of **PB13** (Fig. 5a), the fragment ion at *m/z* 80.0509 was generated by loss of 44 Da (CO_2_) from the molecular ion at *m/z* 124.039, indicating the presence of pyridine ring and carboxyl residue in the chemical structure. Accordingly, nitrobenzene could be removed away from the candidate lists owing to the absence of pyridinering and carboxyl residue in its structure. By comparing our experimental MS/MS spectrum (Fig. 5a) with the MS/MS spectra of the rest 3 candidates in the METLIN database (Fig. 5b–d), **PB13** was reasonably identified as nicotinic acid owing to its excellent match with the MS/MS spectrum in the database. As shown in Fig. 5a, aiding by the fragments pane integrated in the Peakview software, all the major experimental fragment ions of **PB13** were also tentatively assigned to certain chemical structures, with good mass accuracy. It should be noted that the above process could not always guarantee the certain tentative identification. For example, **PB28** and **PB30** were just assigned as a pair of isomers, 5-Pyridoxic acid or 4-Pyridoxic acid, from the interpretation of the MS and MS/MS data. When it comes to this case, published literatures may provide some useful information for further identification. 4-Pyridoxic acid was reported to be increased in the rats with diabetic nephropathy,^33^ which was in consistent with the up-regulation of **PB30** in CDDP-exposed rats. Accordingly, **PB28** and **PB30** were tentatively assigned as 5-Pyridoxic acid and 4-Pyridoxic acid respectively.

**Figure 6:**
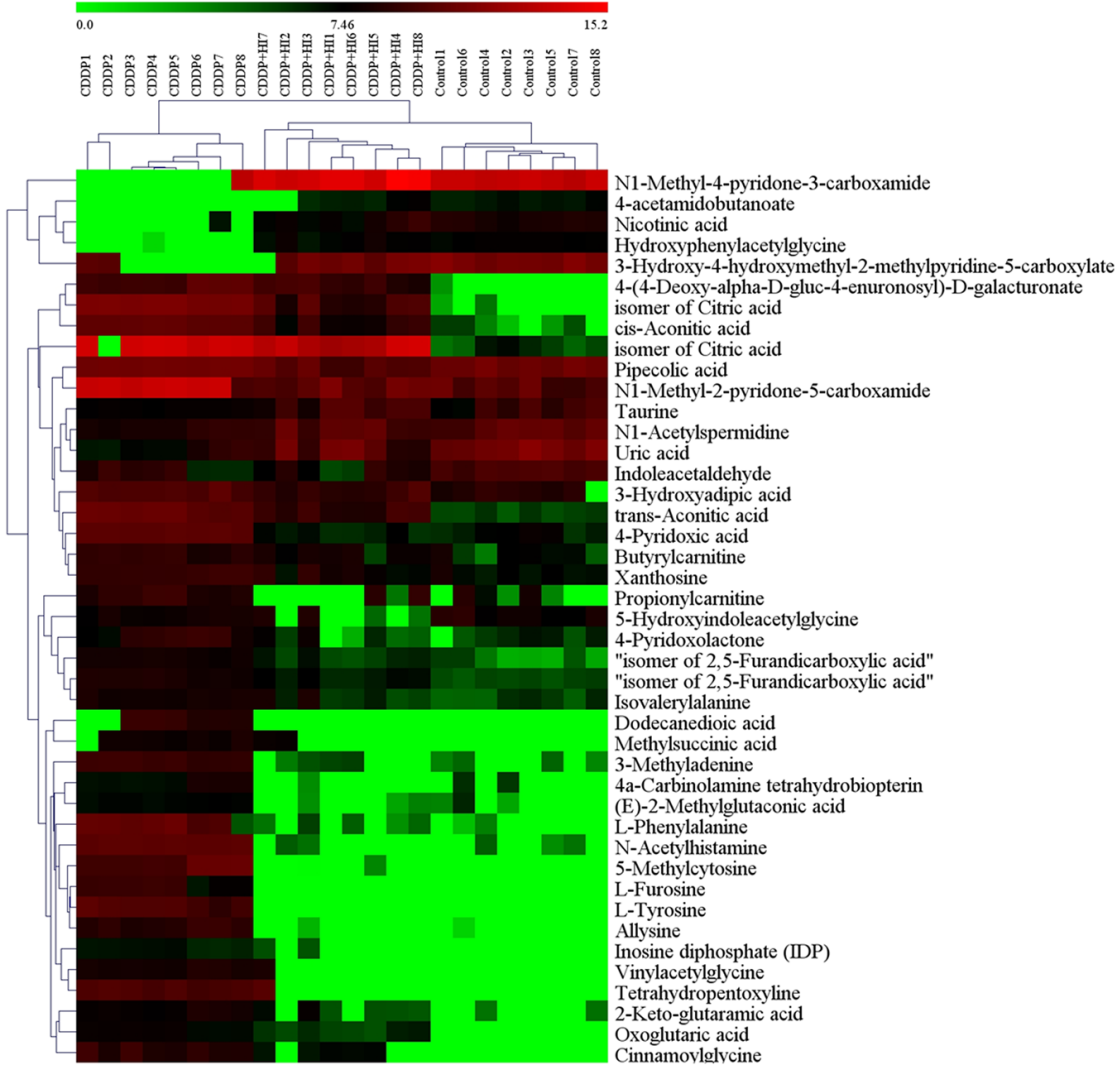
The heatmap and hierarchical cluster analysis of relative contents of 43 potential biomarkers.

Finally, 43 of 176 PBs were reasonably identified using the above steps. Table 1 summarized the general information of all 43 identified PBs, while their Heatmap and HCA results were shown in Fig. 6. The color of Heatmap demonstrated the clear up- or down- regulation of the PBs in 3 groups, while HCA analysis displayed the correlation of the samples (from the sample tree) and the identified PBs (from the gene tree). Group CDDP + HI was closer to Control than CDDP in the structure of sample tree, indicating that HI administration could recover the CDDP-induced abnormality of the identified PBs. This is consistent with the results of PCA analysis (Fig. 3).

**Table 1:** Urine endogenous metabolites identified as potential biomarkers for the protection of HuangQi Injection from CDDP-induced toxicity.

No.	Ion type	m/z	*t*_R_ (min)	Formula	Identity	Trend	CDDP to Control			CDDP to (CDDP + HI)			HMDB	KEGG	^▲^PA number
							p	FC	VIP	p	FC	VIP			
PB1	[M + H]^+^	188.1752	1.65	C_9_H_21_N_3_O	N1-Acetylspermidine	Down	0.000	0.30	2.66	0.000	0.29	2.57	HMDB01276	C00612	21
PB2	[M-H]^−^	124.0082	1.66	C_2_H_7_NO_3_S	Taurine	Down	0.005	0.36	1.58	0.000	0.21	2.98	HMDB00251	C00245	17,18
PB3	[M + H]^+^	146.0446	1.77	C_5_H_7_NO_4_	2-Keto-glutaramic acid	Up	0.000	56.64	1.36	0.000	10.99	1.27	HMDB01552	C00940	12
PB4	[M + H]^+^	353.0709	1.94	C_12_H_16_O_12_	4-(4-Deoxy-alpha-D-gluc-4-enuronosyl)-D-galacturonate	Up	0.000	∞	1.54	0.003	2.36	1.01	METPA0762	C06118	20
	[M-H]^−^	351.0562	1.83			Up	0.000	∞	2.59	0.000	7.60	1.20			
PB5	[M + H]^+^	130.0863	1.92	C_6_H_11_NO_2_	Pipecolic acid	Up	0.003	2.05	1.47	0.000	∞	2.36	HMDB00070	C00408	14
PB6	[M + H]^+^	193.0341	1.92	C_6_H_8_O_7_	isomer of Citric acid	Up	0.000	674.10	4.36	0.000	3.95	3.63	HMDB00094	C00158	6,10,12
	[M-H]^−^	191.0197	1.98			Up	0.000	16.59	14.42	0.000	2.39	12.09			
PB7	[M + H]^+^	429.0221	1.95	C_10_H_14_N_4_O_11_P_2_	Inosine diphosphate	Up	0.000	∞	1.02	0.000	∞	0.98	HMDB03335	C00104	13
PB8	[M + H]^+^	157.0129	1.96	C_6_H_4_O_5_	isomer of 2,5-Furandicarboxylic acid	Up	0.000	11.29	1.50	0.000	4.48	1.34	HMDB04812		25
PB9	[M + H]^+^	175.0235	1.97	C_6_H_6_O_6_	cis-Aconitic acid	Up	0.000	28.44	3.55	0.000	2.80	2.70	HMDB00072	C00417	6,10
PB10	[M + H]^+^	150.0768	2.35	C_6_H_7_N_5_	3-Methyladenine	Up	0.000	50.05	2.10	0.000	15.31	1.98	HMDB11600	C00913	13
PB11	[M + H]^+^	154.0972	2.37	C_7_H_11_N_3_O	N-Acetylhistamine	Up	0.006	2.65	2.04	0.000	134.01	3.05	HMDB13253	C05135	22
PB12	[M + H]^+^	147.0286	2.38	C_5_H_6_O_5_	Oxoglutaric acid	Up	0.000	∞	1.22	0.000	4.96	1.11	HMDB00208	C00026	4,5,6,12
PB13	[M + H]^+^	124.0398	2.39	C_6_H_5_NO_2_	Nicotinic acid	Down	0.000	0.05	1.73	0.000	0.05	1.49	HMDB01488	C00253	3
PB14	[M + H]^+^	169.0355	2.38	C_5_H_4_N_4_O_3_	Uric acid	Down	0.000	0.27	3.00	0.007	0.20	3.24	HMDB00289	C00366	13
	[M-H]^−^	167.0208	2.39			Down	0.000	0.12	4.14	0.003	0.16	3.53			
PB15	[M-H]^−^	191.0201	2.5	C_6_H_8_O_7_	isomer of Citric acid	Up	0.000	188.50	10.41	0.000	2.39	12.09	HMDB00094	C00158	6,10,12
	[M + H]^+^	193.034	2.4			Up	0.000	∞	4.13	0.000	2.86	3.09			
PB16	[M + H]^+^	157.0129	2.51	C_6_H_4_O_5_	isomer of 2,5-Furandicarboxylic acid	Up	0.000	7.18	1.58	0.000	2.72	1.29	HMDB04812		25
PB17	[M + H]^+^	175.0233	2.52	C_6_H_6_O_6_	trans-Aconitic acid	Up	0.000	59.12	3.48	0.000	3.63	2.87	HMDB00958	C02341	6,10
PB18	[M + H]^+^	255.1331	2.72	C_12_H_18_N_2_O_4_	L-Furosine	Up	0.000	65.76	1.74	0.000	∞	1.68	HMDB29390		23
PB19	[M + H]^+^	126.0663	3.02	C_5_H_7_N_3_O	5-Methylcytosine	Up	0.000	∞	2.67	0.000	∞	2.57	HMDB02894	C02376	24
PB20	[M + H]^+^	182.0805	3.03	C_9_H_11_NO_3_	L-Tyrosine	Up	0.000	∞	2.57	0.000	∞	2.46	HMDB00158	C00082	7,2,8,9,1
PB21	[M+H]^+^	240.1085	3.04	C_9_H_13_N_5_O_3_	4a-Carbinolamine tetrahydrobiopterin	Up	0.000	∞	1.17	0.000	∞	1.13	HMDB02215	C00268	15
PB22	[M+H]^+^	145.049	3.42	C_6_H_8_O_4_	(E)-2-Methylglutaconic acid	Up	0.000	101.92	1.13	0.000	8.28	1.00	HMDB02266		25
PB23	[M+H]^+^	146.0811	3.48	C_6_H_11_NO_3_	4-Acetamidobutanoic acid	Down	0.000	0.00	1.18	0.000	0.00	1.04	HMDB03681	C02946	16
	[M-H]^−^	144.0665	3.44			Down	0.000	0.00	1.09	0.001	0.00	1.12	HMDB03681		
PB24	[M-H]^−^	161.0457	3.62	C_6_H_10_O_5_	3-Hydroxyadipic acid	Up	0.000	3.16	2.41	0.000	2.36	2.42	HMDB00345		25
PB25	[M+H]^+^	231.1587	3.79	C_12_H_22_O_4_	Dodecanedioic acid	Up	0.000	∞	1.83	0.000	∞	1.74	HMDB00623	C02678	25
PB26	[M+H]^+^	210.0753	3.86	C_10_H_11_NO_4_	Hydroxyphenylacetylglycine	Down	0.000	0.00	1.22	0.000	0.00	1.38	HMDB00735	C05596	1
PB27	[M+H]^+^	153.0659	4.06	C_7_H_8_N_2_O_2_	N1-Methyl-4-pyridone-3-carboxamide	Down	0.000	0.00	10.43	0.000	0.05	13.53	HMDB04194	C05843	3
PB28	[M-H]^−^	182.0464	4.18	C_8_H_9_NO_4_	5-Pyridoxic acid	Down	0.000	0.00	4.66	0.001	0.23	4.05	METPA0540	C04773	11
PB29	[M+H]^+^	218.138	4.22	C_10_H_20_NO_4_	Propionylcarnitine	Up	0.000	7.86	1.62	0.000	70.67	1.77	HMDB00824	C03017	25
PB30	[M+H]^+^	184.0604	4.53	C_8_H_9_NO_4_	4-Pyridoxic acid	Up	0.001	2.70	2.30	0.003	4.18	2.33	HMDB00017	C00847	11
PB31	[M+H]^+^	153.0657	4.57	C_7_H_8_N_2_O_2_	N1-Methyl-2-pyridone-5-carboxamide	Up	0.000	11.24	9.54	0.000	8.09	8.01	HMDB04193	C05842	3
PB32	[M+H]^+^	166.0494	4.74	C_8_H_7_NO_3_	4-Pyridoxolactone	Up	0.000	4.43	1.52	0.000	43.95	1.26	HMDB03454	C00971	11
PB33	[M+H]^+^	160.0755	5.14	C_10_H_9_NO	Indoleacetaldehyde	Down	0.008	0.43	1.59	0.002	0.03	1.38	HMDB01190	C00637	19
PB34	[M+H]^+^	144.0649	5.01	C_6_H_9_NO_3_	Vinylacetylglycine	Up	0.000	10.27	1.04	0.000	7.83	0.98	HMDB00894		25
	[M-H]^−^	142.0502	5.35			Up	0.000	∞	1.56	0.000	6.42	1.51			
PB35	[M+H]^+^	146.081	5.66	C_6_H_11_NO_3_	Allysine	Up	0.000	∞	1.26	0.000	8.27	1.10	HMDB01263	C01475	11
	[M-H]^−^	144.0668	5.74			Up	0.000	75.35	3.10	0.000	6.40	3.05			
PB36	[M+H]^+^	166.0862	5.81	C_9_H_11_NO_2_	L-Phenylalanine	Up	0.000	3.58	2.37	0.000	177.28	2.67	HMDB00159	C00079	7
PB37	[M-H]^−^	365.1348	5.88	C_17_H_22_N_2_O_7_	Tetrahydropentoxyline	Up	0.000	∞	2.78	0.000	5.87	2.65	HMDB29992		19
PB38	[M-H]^−^	131.0353	6.82	C_5_H_8_O_4_	Methylsuccinic acid	Up	0.000	∞	1.43	0.005	2.25	1.08	HMDB01844	C08645	26
PB39	[M+H]^+^	249.0867	7.46	C_12_H_12_N_2_O_4_	5-Hydroxyindoleacetylglycine	Up	0.000	18.57	1.56	0.000	41.86	1.39	HMDB04185	C05832	19
PB40	[M+H]^+^	232.154	7.54	C_11_H_22_NO_4_	Butyrylcarnitine	Up	0.001	2.90	1.82	0.010	2.32	1.16	HMDB02013	C02862	25
PB41	[M+H]^+^	285.0843	9.24	C_10_H_12_N_4_O_6_	Xanthosine	Up	0.000	3.45	1.75	0.000	2.70	1.58	HMDB00299	C01762	13
PB42	[M-H]^−^	172.0981	10.48	C_8_H_15_NO_3_	Isovalerylalanine	Up	0.000	3.56	2.00	0.000	2.24	2.00	HMDB00747		25
	[M+H]^+^	174.1124	10.45			Up	0.000	5.46	1.44	0.000	4.04	1.33			
PB43	[M+H]^+^	206.0816	10.96	C_11_H_11_NO_3_	Cinnamoylglycine	Up	0.000	∞	1.33	0.009	2.89	1.18	HMDB11621		25
	[M-H]^−^	204.0666	10.95			Up	0.000	∞	1.79	0.000	5.01	1.73			

^▲^PA1: Tyrosine metabolism; PA2: Ubiquinone and other terpenoid-quinone biosynthesis; PA3: Nicotinate and nicotinamide metabolism; PA4: D-Glutamine and D-glutamate metabolism; PA5: Butanoate metabolism; PA6: tricarboxylic acid cycle (TCA cycle); PA7: Phenylalanine, tyrosine and tryptophan biosynthesis; PA8: Phenylalanine metabolism; PA9: Aminoacyl-tRNA biosynthesis; PA10: Glyoxylate and dicarboxylate metabolism; PA11: Vitamin B6 metabolism; PA12: Alanine, aspartate and glutamate metabolism; PA13: Purine metabolism; PA14: Lysine degradation; PA15: Folate biosynthesis; PA16: Arginine and proline metabolism; PA17: Taurine and hypotaurine metabolism; PA18: Primary bile acid biosynthesis; PA19: Tryptophan metabolism; PA20: Pentose and glucuronate interconversions; PA21: polyamine metabolism; PA22: Histidine metabolism; PA23: lysine metabolism; PA24: Pyrimidine metabolism; PA25: Fatty acid metabolism; PA26: isoleucine catabolism.

### Metabolic pathway analysis

The dataset of the 43 identified PBs including HMDB number and MSTUS-normalized relative peak intensity were imported into the web-based MetaboAnalyst 3.0 system for Pathway Analysis and visualization. As a result, 26 of the identified PBs were found to be associated with 19 metabolic pathways (PA1-PA19 shown in Table 1), 7 of which including Phenylalanine, tyrosine and tryptophan biosynthesis (PA7), Glyoxylate and dicarboxylate metabolism (PA10), Taurine and hypotaurine metabolism (PA17), Phenylalanine metabolism (PA8), tricarboxylic acid cycle (TCA cycle, PA6), Tyrosine metabolism (PA1), and Alanine, aspartate and glutamate metabolism (PA12) (Fig. S2) are the significantly changed pathways (*p* > 0.06) associated with the toxicity-attenuation effect of HI. In addition, according to the related publication literatures, another 8 pathways (PA20-PA26) associated with the other 17 PBs were also summarized in Table 1.

## Discussion

The action mechanism of intervention effect of TCMs on nephrotoxicity was very complex and not yet fully understood. Metabolomics offered a promising way to understand the action mechanism of therapeutic effect of TCMs especially at the holistic metabolic level^33–36^. As for the CDDP-induced nephrotoxicity, previous urinary metabolomics studies have revealed that several major metabolic pathways including TCA cycle, and amino acids metabolism were all disturbed by single administration of CDDP^5, 6, 37, 38^. However, toxicity induced by a single dose of CDDP normally recovered back into normal in a short period, which was not suitable for the relatively long-term investigation on the toxicity-attenuation effect of HI. Therefore, in this study, repeated CDDP administration was employed to prolong the nephrotoxicity duration, with the purpose of guaranteeing long enough period of HI successive administration to exert its toxicity-attenuation effect. The dosage and interval of CDDP were optimized to obtain remarkable nephrotoxicity and good tolerance in our preliminary test, and finally, five times of CDDP administration on day 1, 3, 7, 11 and 14, with the daily dosage of 2.5 mg/kg, were adopted in this study. All the rats were well tolerated during the whole experimental period, while the results from both the general pharmacological assessment (Fig. 2) and sample clustering in PCA score plots (Fig. 3) clearly demonstrated that the presence of noticeable toxicity in Group CDDP compared to Group Control, as well as the protection or attenuation effect of HI. This indicated that our protocol of CDDP repeated administration is feasible and suitable for studying the long-term pharmacological effect of Herbal preparations such as HI.

**Figure 7:**
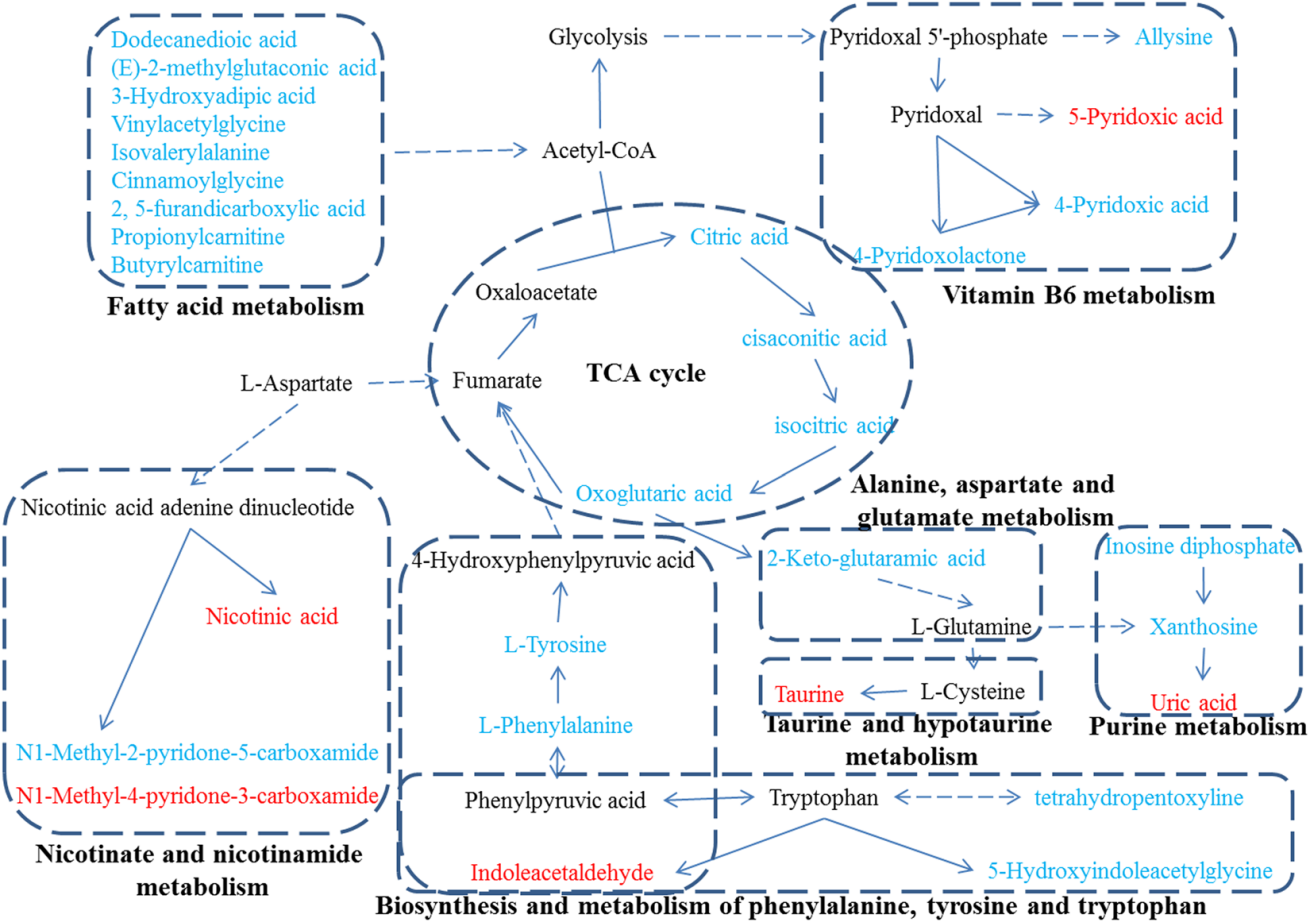
The metabolic pathway networks of potential biomarkers (PBs) in response to the toxicity-attenuation effects of HuangQi Injections on cisplatin-exposed rats. The identified PBs are labeled in blue (up-regulated in Group CDDP) and red (down-regulated in Group CDDP), respectively.

In this study, 43 urinary endogenous metabolites were identified as the toxicity-attenuation PBs of HI administration, which were involved in 26 altered metabolic pathways or physiological functions (Table 1). It can be seen that the relative urinary levels of most identified PBs (34/43) were increased after CDDP exposure, while repeated HI administration could restore this abnormal increase. Pathway analysis of all the identified PBs dataset by MetaboAnalyst revealed that 10 identified PBs were attributed to the 7 significantly altered metabolic pathways (Fig. S2). Besides, it should be noted that there were another 5 pathways (PA 3, 11, 13, 19, and 25) assigned by no less than 3 identified PBs. Monitoring changes in these PBs and related pathway networks (Fig. 7) could highlight on the complex mechanism of CDDP-induced toxicity, as well as the protective effect of HI.

Amino acids (AAs) have important functions in both nutrition and health. There is growing recognition that besides their role as building blocks of proteins and polypeptides, some AAs regulate key metabolic pathways that are necessary for maintenance, growth, reproduction, and immunity^39^. It has been reported that AAs metabolism including phenylalanine, glutamine, targinine, tryptophan, taurine, alanine and cysteine were significantly altered in patients with chronic kidney disease^40–42^. As shown in Table 1, many PBs involved in the metabolism of phenylalanine, tyrosine, tryptophan, alanine, aspartate, glutamate, lysine, arginine, proline, taurine and histidine were tentatively identified. Of them, the levels of L-Tyrosine and L-Phenylalanine were significantly increased in Group CDDP, indicating significant changes of phenylalanine-associated pathways (Fig. 7). Similarly, the increase of tetrahydropentoxyline and 5-Hydroxyindoleacetylglycine, as well as the decrease of indoleacetaldehyde, demonstrated that the definite disturbance of tryptophan metabolism induced by CDDP repeated administration. Vitamin B6 was reported to be closely associated with AAs metabolism^43^. The presence of allysine, 4-pyridoxolactone, 4-pyridoxic acid and 5-pyridoxic acid indicated that the balance of vitamin B6 metabolism was disturbed, which would accordingly affect the normal AAs metabolism. Taurine was the end product of metabolic degradation of cysteine. It was considered as the most abundant amino acid in the leukocytes and plays an essential role in mediating normal immune function^44^. The CDDP-induced down-regulation of taurine may hint us the decline of immune function. According to descriptions of HMDB dataset, methylsuccinic acid is a normal urinary metabolite associated with isoleucine catabolism, which was reported to be decreased in rat urine under D-serine-induced nephrotoxicity^45^. However, our results showed that CDDP repeated treatment could lead to the significant increase of methylsuccinic acid, and HI could partly reverse this increase. Overall, repeated CDDP exposure lead to remarkable disturbance of the metabolism of phenylalanine and some functional AAs such as tryptophan, arginine, cysteine, glutamine and proline, while successive HI treatment could effectively restore the balance of AAs metabolism.

Six PBs, inlcluding oxoglutaric acid, pair isomers of citric acid, trans-aconitic acid, cis-aconitic acid, and 2-Keto-glutaramic acid, were found to be associated with TCA cycle. Their urinary levels were all increased significantly (more than 20 fold) after repeated CDDP injection, and could be downregulated remarkably (more than 2.5 fold) by successive HI administration. Previously, urinary metabolomics studies revealed that single-dose CDDP could lead to the upregulation of four important intermediates of TCA cycle including 2-oxoglutarate, pyruvate, valine, and glutamine^1, 46^ which was in consistent with our findings. However, TCA metabolites such as succinate, malate and glutamate were reported to be decreased in rat serum^6^ or urine^47^ after CDDP administration. The higher or lower levels of TCA metabolites may provide some information for elucidating the mechanism of nephrotoxicity and therapy. For example, oxoglutaric acid could be converted to succinate with the generation of NADH. NADH could further convert to NADPH to detoxify the reactive oxygen species (ROS) mediated cellular damages. The accumulation of oxoglutaric acid indicated less efficient conversion of oxoglutaric acid to succinate, leading to less generation of NADH and NADPH, and the subsequent accumulation of ROS-mediated cellular damages^47^. HI could partly correct the abnormal accumulation of oxoglutaric acid to alleviate the ROS-mediated cellular damages by CDDP. Since TCA plays an important role in energy metabolism, the correction of energy metabolism could be one possible therapy mechanism of HI. Furthermore, the identification of four purine metabolites including 3-methyladenine, inosine diphosphate, xanthosine and uric acid demonstrated that purine metabolism, another energy metabolism related pathway, was also disturbed by CDDP and could be corrected by HI. Besides, 3-methyladenine was commonly used as the autophagic inhibitor^48^. Considering that autophagy was reported to increase in renal tissue of CDDP-treated mice^49^, the presence of 3-methyladenine indicated that autophagy may be related to the reno-protection of HI.

Fatty acids are important constituents of cell membranes and the largest energy reserve in the body. The dysregulations of fatty acids including tetracosanoic acid, octadecanamide, docosatrienoic acid, 9-oxooctadecanoic acid, palmitic acid, arachidonic acid and eicosatrienoic acid were demonstrated in both patients with chronic kidney disease and animal models^50–53^. It was reported that single CDDP dose could lead to the accumulation of nonesterified fatty acids and triglycerides in serum, urine and kidney tissue, while the activation of intracellular calcium-independent phospholipase A2 (PA2) and the inhibition of mitochondrial fatty acid oxidation were considered as the potential mechanisms responsible for the accumulation of free fatty acids^5^. Similar findings were observed in our current study. Eight free fatty acids including dodecanedioic acid, (E)−2-methylglutaconic acid, 3-Hydroxyadipic acid, vinylacetylglycine, isovalerylalanine, cinnamoylglycine and a pair isomer of 2,5-furandicarboxylic acid were identified as PBs, with their levels increased in Group CDDP and recovered in Group CDDP + HI, indicating that the activation of fatty acid oxidation and the inhibition of PA2 may be the potential mechanisms of the toxicity-attenuation effect of HI. Carnitine is an essential cofactor required to transport fatty acids into the inner mitochondrial matrix to provide energy through β-oxidation. The decrease of propionylcarnitine, a product of the enzymatic esterification of carnitine, was observed in mousecrystal-induced kidney injury^54^. Unlike that, in our current study, the levels of propionylcarnitine and Butyrylcarnitinewere significantly increased after CDDP administration, and HI could effectively restore this up-regulation. These findings further demonstrated that the correction of fatty acid metabolism disorder would be one therapy mechanism of HI.

## Conclusion

Repeated treatment with HI could alleviate CDDP-induced nephrotoxicity, and restore the abnormality of urinary metabolic profile caused by CDDP exposure. According to the tentative identification of 43 PBs, the attenuation effect of HI on CDDP-induced toxicity may be associated with the restoration of the disturbance in the TCA cycle and metabolisms of amino acid, fatty acid, vitamin B6 and purine. The present study provided reliable evidence for the protective effect of HI on CDDP-induced toxicity. Further investigations on the level of tumor-bearing rats are still needed to clarify whether HI is a promising preparation combined with CDDP in cancer therapy.

## Electronic supplementary material

Supplementary tables and Figures
