# Supplementary material for: Urinary metabolomics reveals the therapeutic effect of HuangQi Injections in cisplatin-induced nephrotoxic rats
Source: Sci Rep. 2017 Jun 15;7:3619. doi: 10.1038/s41598-017-03249-z (PMC5472607; doi:10.1038/s41598-017-03249-z)
Supplement: Supplementary file 1 — Supplementary tables and Figures [file 41598_2017_3249_MOESM1_ESM.pdf]

Title Page

Urinary metabolomics reveals the therapeutic effect of HuangQi Injections in cisplatin-induced nephrotoxic rats

Chang-Yin Li[†*a], Hui-Ting Song[†a], Xiao-Xiao Wang[a], Yao-Yao Wan[b], Xuan-Sheng Ding[b], Shi-Jia Liu[a], Guo-Liang Dai[a], Yue-Heng Liu[c] & Wen-Zheng Ju*[a]

Table S1 Stability of m/z, t_R and intensity for the 16 typical ions in pooled QC sample in both positive (n=5) and negative (n=5) ion modes.

No.	m/z			t_R			Intensity		
	Mean	Δd	ppm	Max	Min	Δd	Mean	SD	RSD
P1	114.0668	0.000400	3.51	1.75	1.74	0.01	1837809	92633	5.04
P2	166.0721	0.000620	3.73	2.68	2.57	0.11	558665	24043	4.30
P3	245.1604	0.001290	5.26	3.14	3.00	0.14	342986	9326	2.72
P4	153.0657	0.000790	5.16	4.19	3.98	0.21	250746	13633	5.44
P5	367.1495	0.001970	5.37	5.49	5.29	0.20	28783	2845	9.88
P6	158.0810	0.000850	5.38	6.85	6.72	0.13	90857	7772	8.55
P7	297.1440	0.001490	5.01	7.44	7.37	0.07	254875	7693	3.02
P8	338.0867	0.001730	5.12	8.87	8.85	0.02	439183	32290	7.35
P9	194.0810	0.000820	4.23	9.64	9.63	0.01	2195841	41134	1.87
P10	164.0708	0.000860	5.24	10.28	10.27	0.01	944805	21582	2.28
P11	255.0649	0.001490	5.84	11.78	11.77	0.01	413729	25333	6.12
P12	255.1222	0.001000	3.92	12.74	12.73	0.01	107889	5271	4.89
P13	246.2426	0.001370	5.56	13.37	13.36	0.01	506225	15965	3.15
P14	274.2739	0.001520	5.54	14.75	14.75	0.00	4148105	364786	8.79
P15	302.3050	0.001470	4.86	15.92	15.90	0.02	1817394	65473	3.60
P16	279.1586	0.001420	5.09	16.40	16.30	0.10	41701	3041	7.29
Pmax			5.84			0.21			9.88
Pmin			3.51			0.00			1.87
N1	191.0202	0.000340	1.78	1.91	1.90	0.01	2265921	132842	5.86
N2	191.0198	0.000140	0.73	2.41	2.39	0.02	2086223	112336	5.38
N3	161.0458	0.000220	1.37	3.28	3.20	0.08	32264	1670	5.18
N4	182.0462	0.000190	1.04	4.34	4.28	0.06	53126	3956	7.45
N5	144.0671	0.000110	0.76	5.35	5.30	0.05	44256	1963	4.44
N6	227.9974	0.000130	0.57	6.22	6.07	0.15	57467	2768	4.82
N7	218.1038	0.000640	2.93	7.10	7.05	0.05	93844	3775	4.02
N8	212.0026	0.000250	1.18	8.78	8.76	0.02	933582	31248	3.35
N9	192.0671	0.000210	1.09	9.65	9.63	0.02	2112986	63674	3.01
N10	162.0567	0.000320	1.97	10.30	10.28	0.02	412692	38993	9.45
N11	253.0509	0.000670	2.65	11.79	11.79	0.00	427578	19587	4.58
N12	269.0458	0.000910	3.38	12.58	12.58	0.00	308753	28621	9.27
N13	401.1236	0.002620	6.53	13.08	13.07	0.01	77914	3355	4.31
N14	293.1760	0.001090	3.72	14.17	14.16	0.01	67227	3826	5.69
N15	407.2800	0.002120	5.21	15.17	15.16	0.01	68709	6541	9.52
N16	391.2852	0.002730	6.98	16.06	16.05	0.01	43443	3855	8.87
Nmax			6.98			0.15			9.52
Nmin			0.57			0.00			3.01

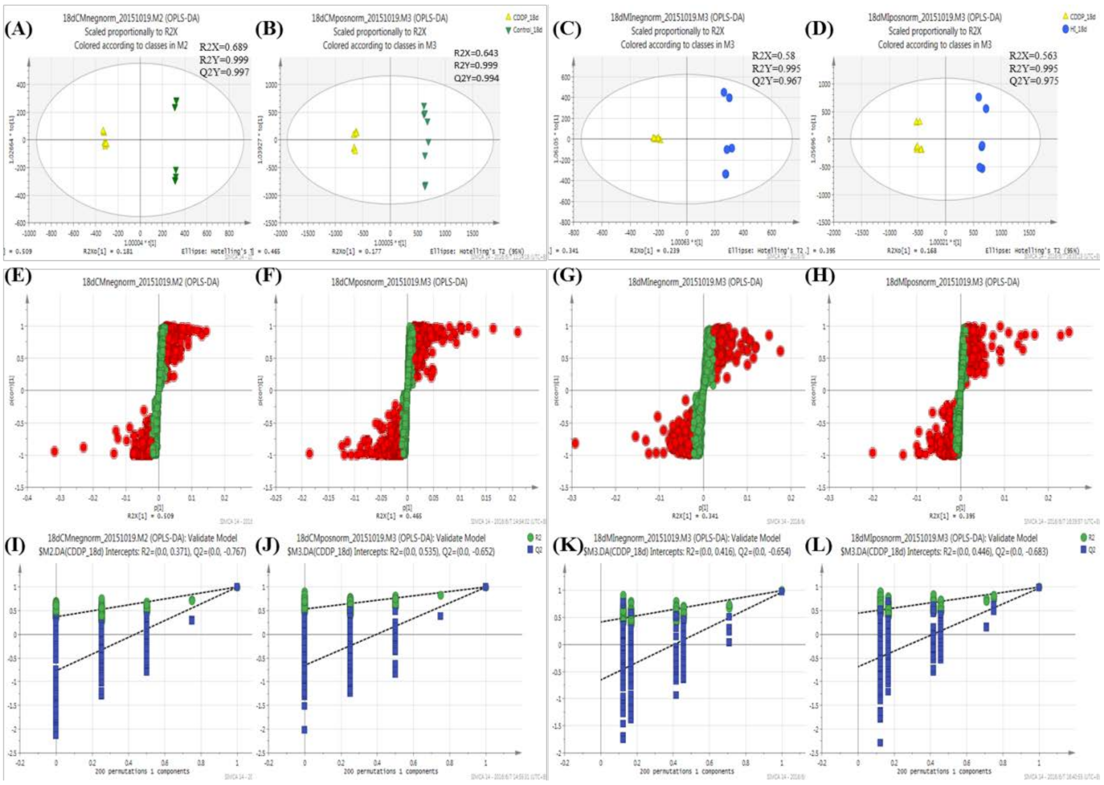

Figure S1 The results of OPLS-DA analysis of LC-MS data in rat urine samples including:(A) score plot between Group CDDP and Control in negative ion mode, (B) score plot between Group CDDP and Control in positive ion mode, (C) score plot between Group CDDP and HI in negative ion mode, (D) score plot between Group CDDP and HI in positive ion mode, (E) S-plot between Group CDDP and Control in negative ion mode, (F) S-plot between Group CDDP and Control in positive ion mode, (G) S-plot between Group CDDP and HI in negative ion mode, (H) S-plot between Group CDDP and HI in positive ion mode, (I) chance permutation test between Group CDDP and Control in negative ion mode, (J) chance permutation test between Group CDDP and Control in positive ion mode, (K) chance permutation test between Group CDDP and HI in negative ion mode, (L) chance permutation test between Group CDDP and HI in positive ion mode,

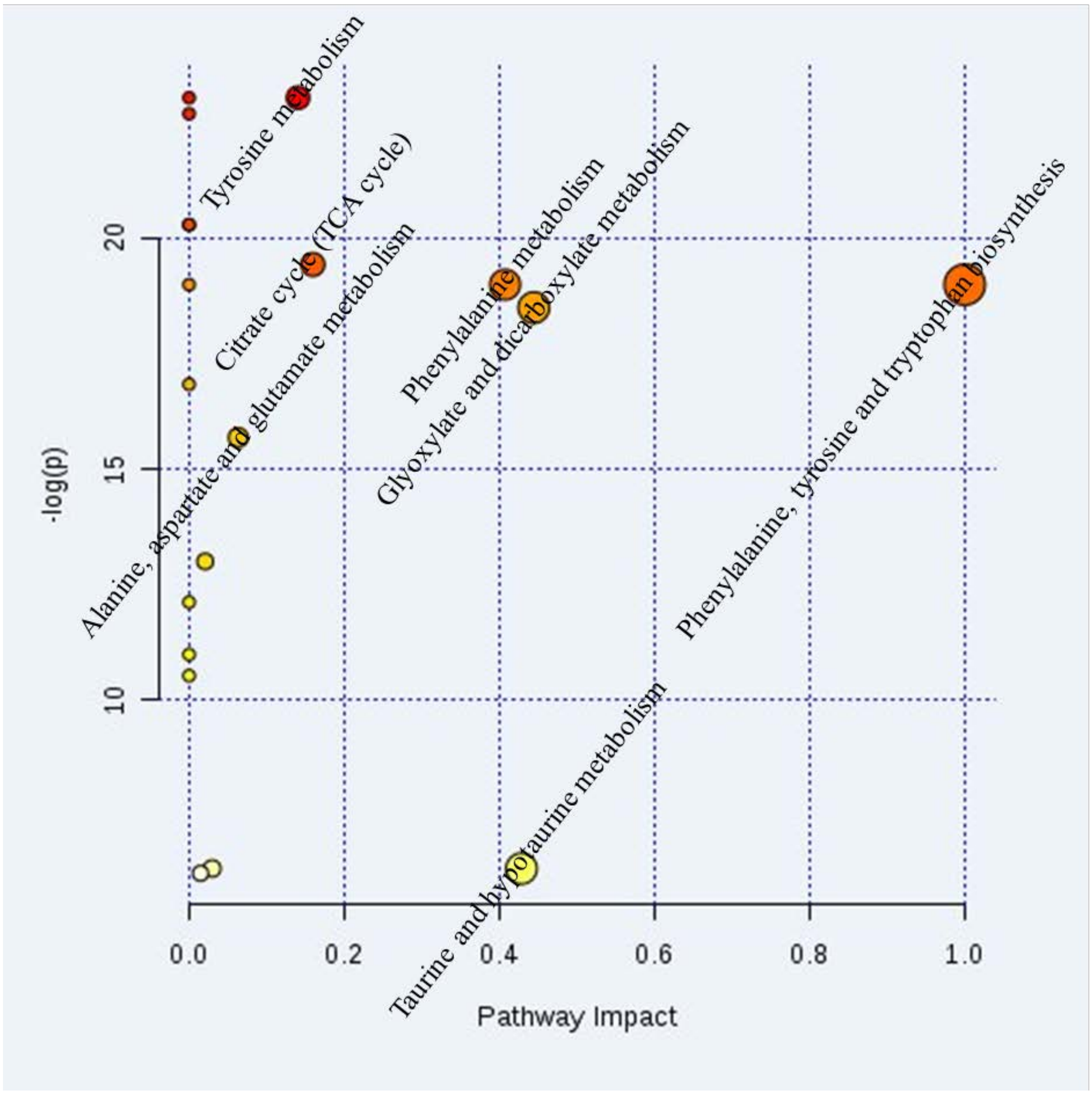

Figure S2 The altered metabolic pathways in rats associated with the toxicity-attenuation effect of HuangQi Injection.
